# Removal of Naso-Pharyngeal Polypus

**Published:** 1882-06

**Authors:** R. F. Weir


### ARTICLE V.

### *Removal of Naso-Pharyngeal Polypus by Nelaton's Operation—Death.*

Dr. Robert F. Weir related the history of an operation for removal of a naso pharyngeal polypus, which had some points of extreme interest.

The patient was a boy, aged eight. A year ago he had had diphtheria, and this was followed by the "snuffles." This symptom did not attract attention for some time. It was at last noticed that he could not sleep in the ordinary position. He then began to have nasal hemorrhages.

He was taken first to a quack who blew powders up his nose. He then went to a physician in Newark, who tried to remove the tumor, which hung down just in view, by splitting the soft palate after Manne's method. The child developed some alarming symptoms from hemorrhage and shock, and was resuscitated with difficulty. Recently he was sent to Dr. Lefferts, of this city, who turned the case over to Dr. Weir.

The child was then in hardly a fair condition of health, but no delay in order to improve him was allowable on account of the recurring hemorrhage and disturbed sleep.

An examination showed the tumor to have encroached anteriorly in the nostrils, but not entirely to have occluded them. It also hung down nearly to the base of the tongue. The tumor was thought by digital examination to be attached to the base of the basilar process of the occipital bone.

The points to be determined before operating were: (1) the kind of operation; (2) the question of preliminary tracheotomy.

*First.*—The experience under the first head, in this city, was embraced in the cases of Dr. Sands and Dr. Peters. They had adopted the plan of taking out the lower portion of the upper jaw, as suggested by Maisonneuve, and thus effecting a broad entrance to the lateral aspect of the base of the basilar process. This operation, though a good one for large tumors, would involve an unnecessary disturbance of the parts in the present case. Vallet's operation also did not seem advisable, though in it less destruction of the bony parts took place, with an efficient lateral exposure. The speaker, therefore, in this instance, as the roof of the mouth was a wide one, determined upon that devised by Nelaton, which consisted in cutting through the soft palate, then removing a large portion of the hard palate along with part of the vomer. [This was demonstrated upon a skull, as was also bony sections of Vallet and Maisonneuve.]

This method exposed the basilar process and nasal fossæ completely, and was, he thought, best adapted for removal of tumors of moderate size. It had been done, according to Robin-Masse, seventeen times with only three deaths. This as well as the other operations were divised with the idea of having a permanent opening leading to the basilar process. The osteoplastic operations should not be entertained by the surgeon, because of the well-known tendency of such tumor to return, which demanded a number of secondary operations often extending over years.

*Second.*—The second point was that regarding the advisability of doing a preliminary tracheotomy.

The speaker had studied the question carefully, and had finally concluded not to do this. In Dr. Sand's case, however, it was not done, and the hemorrhage was easily controlled, and trachea saved from blood intrusion by keeping the head well forward, with the patient only under partial anæsthesia. This plan was somewhat troublesome, but effective. In Dr. Peters' case it was done with a satisfactory result. Notwithstanding this, Dr. Weir was influenced in his decision in part by his belief that tracheotomy was not the comparatively safe operation often supposed. He had found in support of this view that, in ninety-three cases of tracheotomy performed for the early removal of foreign bodies in the air passages, there had been twenty-six deaths. On the other hand, however, the risk that accrued from blood entering the trachea, etc., was not to be statistically estimated. He believed now that his decision not to perform tracheotomy was an error of judgment. This would be apparent from the details of the operation, which were as follows:

The patient was anæstheized and the head thrown strongly backward over a hard pillow (Rose's position) so far that blood would not run into the trachea, and supported on a sand-bag. The operation was then begun, with the mouth held open by Dr. Weir's mouth-gag: the soft parts of the hard palate were cut in the median line and stripped from the bone, and the latter divided with a chisel on each side from the level of first bicuspid tooth and transversely at this point, so that a space nearly an inch wide was made, affording a complete view of the tumor antero-posteriorly, with no difficulty except quite free venous oozing, some which came from a wound of the tumor itself.

At this moment the patient suddenly began to gasp and choke. The speaker could not say whether blood had been sucked into the trachea or not. The asphyxia was so great that tracheotomy was at once performed with a single cut, a tube inserted, when the patient revived. Dr. Weir, however, noticed, when he performed tracheotomy, what he considered an important fact in connectian with Rose's method, and which a subsequent experience, in two other operations in which the plan was adopted, has confirmed, that the trachea was so stretched that the anterior and posterior walls were very close together, and in the first incision the posterior wall was nearly cut through. It seemed to him, therefore, very probable that this stretching and compression of the trachea, more possible in the young subject, had something to do with the bad symptoms.

The tumor was then readily removed without any difficulty and without much loss of blood. The child was, however, much prostrated, but rallied, and at the close of the cauterization of the narrow attachments of the tumor, was apparently doing well. He was then removed to the ward. In a few moments word was sent up that the child was sinking. Death occurred so suddenly that arterial transfusion, which was contemplated, could not be resorted to. The tumor was attached at the usual place of such growths, on the basilar process. It measured $1\frac{3}{4}$ by $1\frac{3}{8}$ by $1\frac{1}{8}$ inches, and weighed 17 grammes, or over half an ounce. Dr. G. L. Peabody, pathologist of the New York Hospital, pronounced it a fibro-sarcoma. It had a few mucous cells, and probably originated as a myxo sarcoma.—*Practitioners' Society of N. Y., in Med. Record.*

